# Scientific misconduct, questionable research practices, and research climate: empirical approaches to integrity problems

**DOI:** 10.1186/s41073-026-00214-1

**Published:** 2026-06-22

**Authors:** Serhii Nazarovets

**Affiliations:** Borys Grinchenko Kyiv Metropolitan University, 18/2 Bulvarno-Kudriavska Str., Kyiv, 04053 Ukraine

**Keywords:** Scientific misconduct, Questionable research practices, Research climate, Research integrity, Scoping review

## Abstract

Empirical research on scientific misconduct and questionable research practices (QRPs) has increasingly adopted a systemic perspective, emphasising research climate, supervision, and organisational conditions. This PRISMA-guided scoping review synthesises 229 survey- and interview-based studies published between 2016 and 2025 to examine how misconduct and QRPs are empirically operationalised in perception- and climate-oriented research. Rather than aiming to estimate prevalence, the review focuses on the concepts, instruments, and methodological choices through which integrity problems are rendered measurable. The findings show that aggregate harm is rarely defined explicitly and is instead inferred from perceived prevalence and severity of practices. Severe violations dominate measurement frameworks, while routine, relational, and structurally induced practices remain weakly captured. Methodological patterns further shape what becomes empirically visible, highlighting the need for approaches that better integrate individual perceptions with institutional conditions when assessing integrity risks.

## Background

### From individual misconduct to systemic conditions

Public and institutional responses to research misconduct have long been dominated by a focus on identifiable offenders and visible violations. Predatory journals, paper mills, fabricated data, and high-profile retractions are typically addressed through exposure, sanctions, and tighter control mechanisms [3, 8, 25, 26]. While such actions are important, they frame misconduct primarily as a problem of individual wrongdoing and deviant actors, implicitly separating integrity failures from the ordinary conditions of research work.

This individualised framing has increasingly been questioned. Commenting on predatory publishing, Siler et al. [32] famously compared it to the Hydra of Greek mythology: when one head is cut off, new ones quickly emerge. The metaphor captures a simple but powerful insight. Reactive and punitive measures may suppress specific manifestations of misconduct, but they do little to address the incentive structures, evaluation systems, and publication pressures that continually generate new problematic practices [22, 35]. As long as these underlying conditions remain in place, integrity problems are likely to reappear in adapted forms.

A similar limitation characterises early empirical attempts to study misconduct through prevalence estimates [9, 10]. Surveys asking how many researchers commit fabrication, falsification, or plagiarism (FFP) promised clarity, but delivered ambiguous results [9, 12]. Direct questions about severe violations are highly sensitive to social desirability and fear of consequences, leading to systematic underreporting [16, 21]. Moreover, reducing misconduct to FFP narrows the analytical lens to rare and extreme cases, while sidelining a broad range of questionable research practices (QRPs) that are more common and often embedded in routine work [7, 14].

In response, the field has gradually shifted its focus. Rather than treating misconduct as an accumulation of individual moral failures, recent empirical research increasingly conceptualises it as a systemic phenomenon. Attention has moved towards researchers’ perceptions of acceptable practice, shared norms within teams and disciplines, and features of the research environment that shape everyday decision-making [14]. This indicates that integrity failures are not anomalies at the margins of science, but recurring outcomes of how research is organised, evaluated, and rewarded.

This shift has important implications for how misconduct and QRPs are studied. Instead of asking how often rules are broken, recent work asks how problematic practices are normalised, tolerated, or discouraged within specific research climates [28, 38]. It is this move, from individual acts to systemic conditions, that provides the starting point for the present review.

Importantly, framing misconduct as a systemic problem does not imply moral neutrality or diminished responsibility of individual actors. Practices such as data fabrication, contract cheating, the purchase of authorship, or the organisation of paper mills involve deliberate deception and remain unequivocally unethical. This review does not seek to relativise such behaviour or to shift blame away from those who knowingly exploit the system. Rather, the systemic perspective highlights a complementary point: large-scale and persistent forms of organised misconduct can only flourish where institutional incentives, evaluation regimes, and oversight mechanisms fail to constrain them effectively. In healthier research systems, such practices would be far more difficult to sustain, less profitable, and more readily detected.

### Enabling environments and informal regulation

Once misconduct is approached as a systemic phenomenon, the analytical focus shifts from individual intent to the conditions that make problematic practices possible or even routine. Much of the earlier literature sought to explain integrity failures through motives, incentives, or personal shortcomings, often implying that better training or stronger sanctions would be sufficient to prevent misconduct. However, empirical work increasingly suggests that such explanations capture only a small part of the problem.

A key contribution in this respect is Kalichman’s [18] study of research integrity officers, which asked a different question: not why researchers violate rules, but which good research practices are missing when misconduct occurs. The findings point consistently to structural factors such as weak supervision, inadequate mentoring, poor data management routines, and limited oversight. Importantly, many researchers involved in misconduct cases had previously completed ethics training, indicating that awareness of rules does not necessarily translate into responsible practice. From this perspective, misconduct is not so much caused by individual choices as it is enabled by environments that fail to support good research practices in everyday work.

In such environments, integrity problems are often managed informally rather than addressed through formal procedures. When reporting channels are perceived as risky, ineffective, or inaccessible, especially for early-career researchers, concerns tend to circulate through unofficial means. Vaidyanathan et al. [37] describe gossip as a form of informal regulation that functions as a warning system: researchers share reputational information about unreliable collaborators or problematic supervisors in order to protect themselves and others. These mechanisms can impose real social costs and shape collaboration patterns without invoking institutional sanctions.

However, informal regulation has clear limits. Gossip may deter or punish individuals, but it rarely changes the practices or conditions that allowed problems to arise in the first place. It operates opaquely, reinforces hierarchies, and often shields senior actors while exposing junior researchers to greater risk. As a result, such mechanisms can stabilise dysfunctional research environments by containing visible problems without producing organisational learning or reform. Together, these findings highlight how research systems can accommodate misconduct through everyday practices and informal controls, while leaving the underlying enabling conditions largely intact.

### Studying culture before changing it

Empirical studies increasingly show that research integrity is shaped less by the formal presence of rules than by how researchers experience their working environments. Survey-based work by Haven et al. [13] demonstrates that perceptions of research climate explain more variation in self-reported misbehaviour than individual characteristics such as career stage or discipline. Research climate refers to researchers’ shared perceptions of organisational norms, expectations, and everyday practices that shape research behaviour within institutions. Commonly cited risk factors, including publication pressure, do not operate in isolation. Their effects are mediated by local norms of supervision, openness, and mutual support, meaning that similar pressures can lead to very different outcomes depending on the surrounding research climate.

This systemic pattern extends beyond individual institutions. Brooker and Allum [5] report relatively low variation in the prevalence of questionable research practices across organisations, suggesting that integrity problems are not confined to some “bad” institutions. Instead, their findings point to broader, system-level pressures that shape research behaviour across organisational boundaries. In this light, focusing on institutional compliance or isolated cases risks missing the wider context in which questionable practices become normalised.

Evidence from different national settings reinforces this conclusion. Abdelkreem et al. [1], studying Egyptian universities, show that the existence of formal integrity policies does not guarantee their integration into everyday research practice. Weak socialisation into integrity norms, unclear expectations, and limited mentoring emerge as more consequential than the absence of rules. Together, these studies underline that research culture is not an abstract or rhetorical notion, but an empirically observable set of shared expectations and practices that condition how rules are interpreted and enacted.

At the same time, the tools used to study research culture and climate are themselves limited. As Pupovac [30] illustrates through the adaptation of the Survey of Organizational Research Climate, measurement instruments embed normative assumptions about what integrity is and how it should be assessed. Translating such tools across contexts involves conceptual choices that affect what becomes visible and what remains overlooked. On the intervention side, Viđak et al. [36] show that evidence-based approaches to changing research culture remain scarce, with culture often invoked as a goal rather than operationalised as an object of analysis.

These limitations point to a simple but often overlooked implication. Before proposing interventions or policy reforms aimed at improving research culture, it is necessary to understand how culture and climate are currently studied, measured, and interpreted. The present review is grounded in this premise: before changing research culture, we need to examine the empirical approaches through which it has been investigated.

Against this background, the aim of this review is to synthesise how scientific misconduct and questionable research practices are empirically studied in climate- and perception-oriented research, rather than to estimate how frequently such practices occur. The focus is on the concepts, instruments, and methodological choices through which integrity problems are rendered visible, comparable, and analysable in survey- and interview-based studies.

To address this objective, the review is organised around two central research questions.

- RQ1. How is harm associated with scientific misconduct and questionable research practices operationalised in empirical studies?
- RQ2. How do methodological designs and institutional factors, particularly research climate and supervision, shape what becomes empirically visible in studies of research misconduct and questionable research practices?

These central questions are further explored through several related analytical sub-questions:

- Which practices are most consistently identified as harmful to knowledge production?
- Which forms of misbehaviour remain weakly captured or invisible in standard empirical instruments?
- How is supervision conceptualised and measured in survey- and interview-based research?
- What recurring methodological patterns and limitations characterise this body of empirical literature?

To address these questions, we conducted a systematic scoping review of empirical studies published between 2016 and 2025.

The remainder of the paper is structured as follows. The next section describes the PRISMA-guided review methodology, followed by the results addressing the two research questions. The discussion then interprets these findings in relation to institutional explanations of research misconduct.

## Methodology

This study adopts a PRISMA-guided scoping review design to identify, screen, and synthesise empirical research on scientific misconduct and questionable research practices (QRPs). The review follows the core logic of the PRISMA 2020 framework [27], while being adapted for descriptive mapping rather than quantitative synthesis. The PRISMA framework provides a structured protocol for identifying, screening, and transparently reporting the selection of studies in systematic reviews. The aim is to map empirical evidence, research designs, and explanatory frameworks, not to estimate pooled prevalence or effect sizes. This approach was therefore selected as the most appropriate methodological framework.

The literature search was conducted in Scopus and the Web of Science Core Collection, which together provide broad interdisciplinary coverage of research ethics, higher education, and scientific practice. The search targeted peer-reviewed journal articles that explicitly addressed scientific misconduct or questionable research practices and relied on primary empirical data. Search terms related to misconduct and QRPs were combined with terms describing empirical data collection methods (survey, questionnaire, interview, focus group, and mixed methods). To avoid conflating research misconduct with student-level academic dishonesty, records referring to undergraduate populations or student cheating were excluded at the search stage. The search was limited to articles published between 2016 and 2025.

All records retrieved from Scopus and Web of Science were exported and merged into a single dataset. Duplicate records were removed manually using a combination of digital object identifiers (DOIs), titles, and author information. After deduplication, 372 unique records remained and were subjected to title and abstract screening.

Screening was conducted by the author based on predefined inclusion and exclusion criteria. Titles and abstracts were reviewed to assess their relevance to the aims of the study. Only studies reporting original empirical data collected from researchers, academics, editors, or other research-performing staff were retained. Studies focusing exclusively on students, undergraduate education, or academic cheating were excluded, as were theoretical or conceptual papers, editorials, commentaries, and studies that examined misconduct indirectly through retraction databases, bibliometric indicators, or simulation-based approaches without primary empirical input.

During the screening process, records that could not be clearly classified as directly examining scientific misconduct or questionable research practices on the basis of titles and abstracts were excluded. This applied in particular to instrument validation studies, policy-oriented qualitative case studies, and broader research ethics investigations in which misconduct or QRPs were not the primary analytical focus. Only studies in which misconduct or QRPs constituted a central empirical object of analysis were retained for synthesis. For each included study, information was extracted on the country or regional context, research population, methodological design, forms of misconduct or QRPs examined, and the main empirical findings.

The review was restricted to English-language journal articles indexed in Scopus and the Web of Science Core Collection. While this reflects the dominant language of international scholarly communication in this field, it may underrepresent empirical studies published in local languages or national outlets. In addition, most included studies relied on self-reported data, which are susceptible to social desirability and recall biases. These limitations are common in empirical research on scientific misconduct and QRPs and were taken into account in the interpretation of the results.

After title and abstract screening based on predefined inclusion and exclusion criteria, 229 records were retained for synthesis. These studies represent empirical research published between 2016 and 2025 that examines scientific misconduct, questionable research practices, or research integrity using survey-based, interview-based, or mixed-methods designs among academic researchers or research-related stakeholders.

In line with the objectives of a scoping review, the included studies were first subjected to descriptive mapping. This step aimed to characterise the empirical literature rather than to aggregate substantive findings. For each study, we recorded publication year, country or regional context, research population, data collection method, and the types of misconduct or questionable research practices examined. In addition, we coded whether studies focused on prevalence, perceptions, research climate, supervision, or perceived harm.

**Fig. 1:**
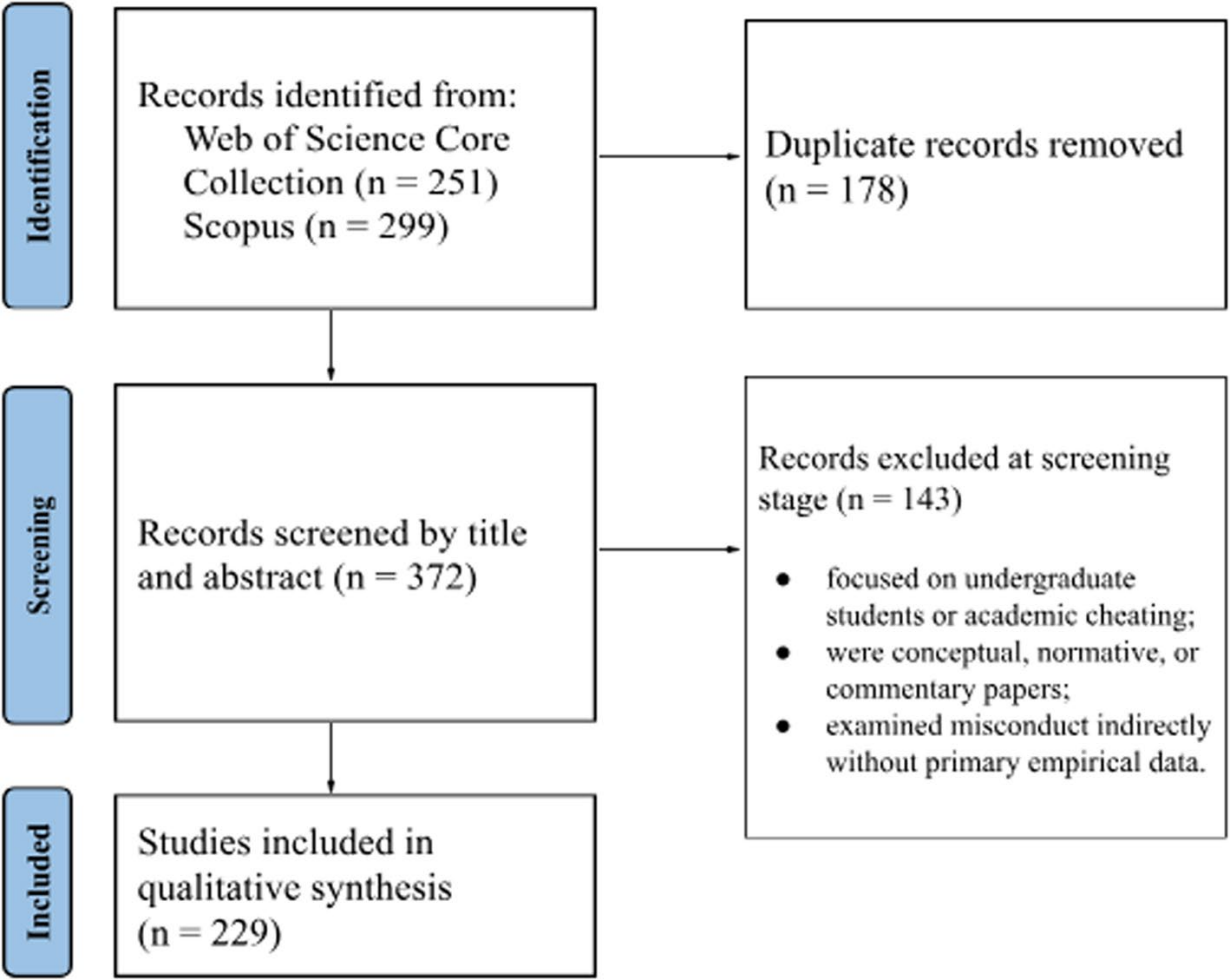
PRISMA-guided flow diagram illustrating the identification, screening, and inclusion of empirical studies on scientific misconduct and questionable research practices (2016–2025)

The descriptive mapping served two purposes. First, it provided an overview of how empirical research on misconduct and QRPs is distributed across contexts, methods, and research populations. Second, it informed the subsequent qualitative synthesis by identifying recurring analytical foci and underrepresented areas (Fig. 1).

## Results

### Characteristics of included studies (descriptive mapping)

**Fig. 2:**
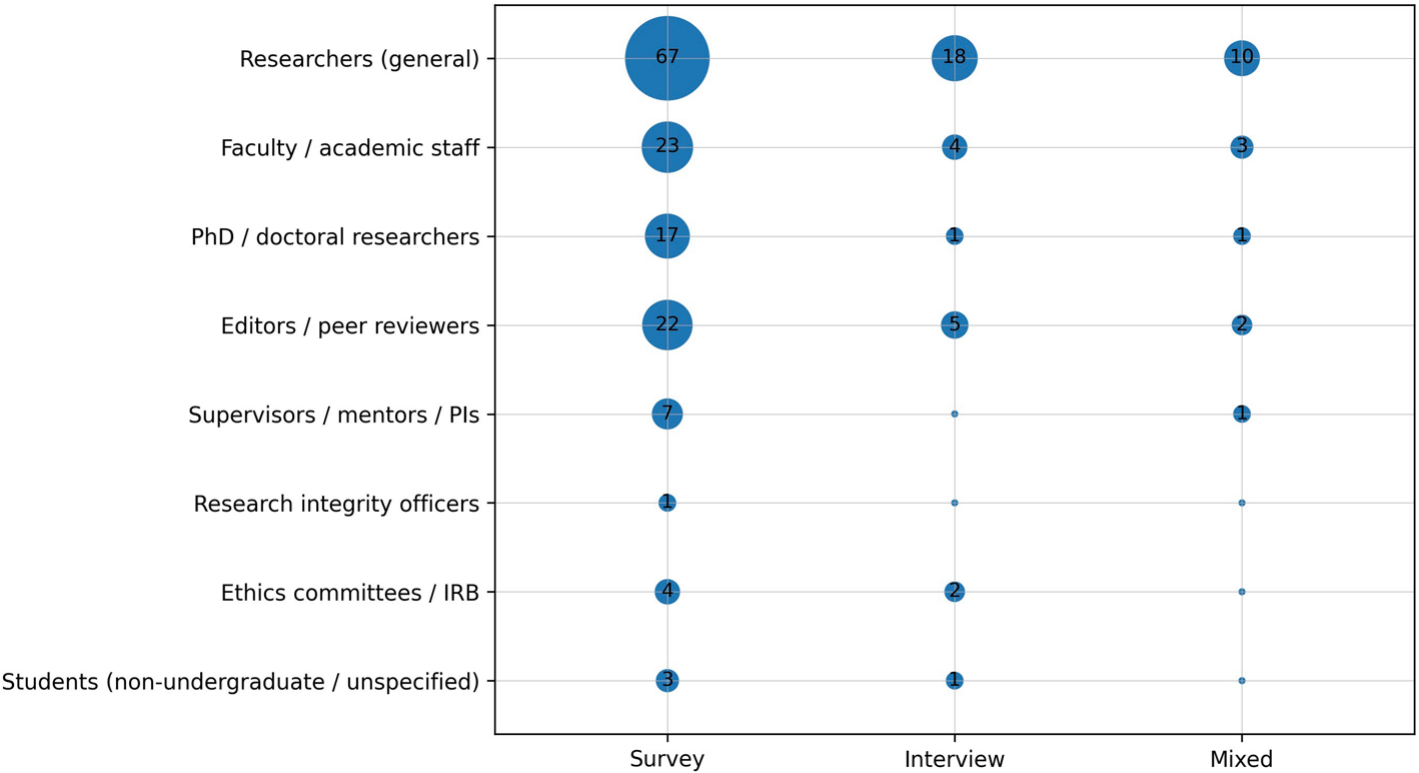
Methods and respondent populations in empirical studies of scientific misconduct and questionable research practices

This section provides a descriptive overview of the empirical literature included in the review. The dataset underlying this descriptive mapping, including structured metadata for all 229 included studies, is openly available via Zenodo (DOI 10.5281/zenodo.18096483). This descriptive overview provides the empirical background for addressing the two research questions formulated in the introduction. The aim is not to summarise substantive findings, but to characterise the corpus in terms of its temporal distribution, geographical coverage, respondent populations, data collection methods, and analytical focus. A complete quantitative mapping of these characteristics is reported in Table 1, while Fig. 2 visualises the relationship between respondent populations and methodological choices.

**Table 1:** Descriptive characteristics of included empirical studies (2016–2025)

Characteristic	Category	n	%
Publication year*	2016	9	3.9
	2017	10	4.4
	2018	26	11.4
	2019	24	10.5
	2020	19	8.3
	2021	24	10.5
	2022	16	7.0
	2023	32	14.0
	2024	38	16.6
	2025	31	13.5
**Geographical context**	Europe	46	20.1
	Asia	24	10.5
	North America	11	4.8
	Middle East & North Africa	9	3.9
	Latin America	3	1.3
	Sub-Saharan Africa	2	0.9
	Oceania	1	0.4
	Multi-region	22	9.6
	Not specified	111	48.5
**Respondent population**	Researchers (general)	101	44.1
	Editors/peer reviewers	30	13.1
	Faculty/academic staff	19	8.3
	PhD/doctoral researchers	19	8.3
	Students (non-undergraduate or unspecified)	18	7.9
	Supervisors/mentors/PIs	11	4.8
	Ethics committees/IRB	6	2.6
	Research integrity officers	4	1.7
	Not specified	21	9.2
**Method**	Survey	148	64.6
	Interview (qualitative)	47	20.5
	Mixed methods	21	9.2
	Not specified	13	5.7
Type of misconduct/QRPs**	Broad misconduct/QRPs	150	65.5
	Authorship-related issues	52	22.7
	Plagiarism/text recycling	51	22.3
	Fabrication/falsification	38	16.6
	Data management/reproducibility	18	7.9
	Selective reporting/publication bias	12	5.2
	Peer review issues	9	3.9
	Conflict of interest	4	1.7
	Ethics approval/IRB issues	4	1.7
	P-hacking/HARKing	3	1.3
**Analytical focus**	Perceptions/attitudes only	56	24.5
	Prevalence only	22	9.6
	Climate/culture only	13	5.7
	Mixed focus incl. climate	47	20.5
	Mixed prevalence & perceptions	38	16.6
	Not specified	53	23.1

^*^ Percentages for year categories are calculated over all 229 records

^**^ Categories are not mutually exclusive and reflect explicit mentions in titles/abstracts

#### Temporal distribution

The 229 included studies were published between 2016 and 2025, with a clearly uneven distribution across the review period (Table 1). Empirical work on scientific misconduct and questionable research practices was present throughout the decade but intensified markedly over time. Only 19 studies (8.3%) were published in 2016–2017, whereas publication activity increased substantially from 2018 onwards. More than half of the corpus appeared after 2020, with the highest concentration observed in the most recent years of the period. This temporal pattern indicates a shift from sporadic early contributions towards a more sustained empirical research agenda.

#### Geographical coverage

Approximately half of the studies explicitly specify a country or regional context in their titles or abstracts, while the remainder do not name a geographical setting. Among studies with an identifiable context, European countries are most frequently represented, followed by Asian and North American settings (Table 1). Other world regions appear far less often and are typically represented by single-country studies. In addition, a subset of publications adopts a multi-country or cross-regional design. The high proportion of studies without an explicit geographical reference suggests that much of the empirical literature implicitly treats misconduct and QRPs as context-independent phenomena, even when national or institutional conditions may differ substantially.

#### Respondent populations

Most empirical studies focus on researchers as a broad and undifferentiated group, without systematically separating career stages or institutional roles (Table 1). Stakeholder groups with formal oversight or governance roles, such as research integrity officers, ethics committee members, and supervisors, are comparatively rare. This imbalance indicates that empirical attention is strongly skewed towards those who perform research, rather than those who regulate, supervise, or adjudicate it.

#### Methods and methodological alignment

Survey-based designs dominate the corpus, accounting for roughly two-thirds of all included studies, while qualitative interview studies and mixed-methods designs are used far less frequently (Table 1). Figure 2 shows that methodological choices are not randomly distributed across respondent populations. Studies of researchers, faculty, and doctoral candidates rely predominantly on surveys, whereas studies involving editors, ethics-related stakeholders, and research integrity officers are more likely to use interviews. Some combinations, such as survey-based research on integrity officers or interview-based studies of large researcher populations, are almost entirely absent. This pattern reflects a strong alignment between method and respondent status, which in turn shapes what kinds of integrity problems become empirically visible.

#### Types of misconduct and analytical focus

Most studies frame their object broadly in terms of scientific misconduct or questionable research practices, often combining multiple behaviours under a single analytical umbrella (Table 1). Authorship-related issues, plagiarism, and fabrication or falsification are the most frequently specified categories, while practices related to peer-review, conflicts of interest, or statistical manipulation appear only sporadically. In terms of analytical focus, studies are more likely to examine perceptions and attitudes than to estimate prevalence, and an increasing share combines these approaches with measures of research climate or organisational culture.

Taken together, the descriptive mapping shows that empirical research on misconduct and QRPs is concentrated in recent years and relies heavily on survey-based methods. It also tends to focus primarily on researchers as respondents, while governance actors and institutional perspectives remain comparatively underexamined. These structural features of the literature are not merely descriptive. They shape what kinds of integrity problems become empirically visible and which dimensions of research systems remain weakly captured in empirical studies.

### Aggregate harm and blind spots in empirical measurement

This section addresses RQ1 by examining how empirical studies operationalise harm associated with scientific misconduct and questionable research practices and which forms of harm remain weakly captured in standard instruments. Rather than evaluating the severity of individual behaviours in normative terms, the focus here is on how harm is rendered measurable across the reviewed literature.

Across the corpus, harm is rarely defined explicitly and is instead inferred by combining information about the perceived frequency of specific practices with assessments of their perceived seriousness or acceptability. In survey-based studies, respondents are often asked how often particular behaviours occur, how problematic they are, or how acceptable they are within their field, thereby implicitly constructing aggregate harm as a combination of perceived prevalence and perceived severity (e.g., [6, 12, 14]). These dimensions are rarely formalised into a single index, but instead remain analytically separate.

Survey instruments typically operationalise harm by presenting respondents with predefined categories of misconduct and asking them to evaluate their severity or acceptability. Within these measurement frameworks, fabrication, falsification, plagiarism, and severe authorship violations appear most prominently as reference categories for defining misconduct (e.g., [9, 16, 29]). However, empirical attention increasingly extends beyond these extreme cases to encompass a broader set of questionable research practices. Selective reporting, inappropriate authorship allocation, and inadequate supervision are often perceived as less severe on an individual level, yet their high reported prevalence positions them as potentially more consequential sources of aggregate harm to knowledge production (e.g., [5, 7, 16]). In this sense, the literature tends to move away from a focus on rare violations towards practices embedded in routine research workflows.

At the same time, the descriptive mapping reveals clear blind spots in how harm is measured. Certain forms of harm are difficult to capture using standardised survey items and therefore appear only marginally in the empirical record. Power asymmetries within research groups, informal coercion in authorship decisions, and the exploitation of early-career researchers are frequently discussed in qualitative and context-specific accounts, but are rarely operationalised as distinct measurable practices in survey-based instruments (e.g., [4, 19]). When they are included, such issues are frequently subsumed under broad or ambiguous categories, which limits their analytical resolution and comparability across studies.

A further blind spot concerns the organisational and systemic dimensions of harm. While some studies adopt a research climate perspective, most instruments still centre on individual behaviours or perceptions rather than on institutional arrangements that enable or constrain those behaviours (e.g., [13, 17, 20, 39]). As a result, harms related to evaluation regimes, funding structures, or publication incentives are typically inferred indirectly rather than measured directly (e.g., [31, 33]). This limits the ability of empirical studies to distinguish between individual-level misbehaviour and structurally induced practices that may be widely tolerated or even implicitly encouraged.

Finally, emerging and organised forms of misconduct, such as paper mills, authorship-for-sale, or coordinated manipulation of peer review, remain largely absent from standard survey instruments, despite their growing visibility in editorial discourse and investigative reporting (e.g., [23, 24]). This temporal lag between the evolution of integrity threats and their measurement contributes to an underestimation of certain types of aggregate harm.

Overall, the empirical literature constructs aggregate harm primarily through what is most readily measurable: self-reported behaviours, perceived norms, and attitudes towards well-defined categories of misconduct. In this sense, harm is not measured directly but constructed through combinations of perceived prevalence, perceived severity, and the availability of survey items capturing specific practices. Practices that are diffuse, relational, or structurally embedded are less consistently captured, creating systematic blind spots. These limitations do not reflect a lack of awareness among researchers, but rather the constraints of prevailing methodological approaches. Understanding these measurement boundaries is essential for interpreting empirical findings on misconduct and for assessing how research climate and supervision are empirically linked to integrity risks. Together, these patterns illustrate how empirical research operationalises harm while simultaneously leaving certain forms of misconduct only partially captured or entirely invisible.

### Enabling conditions: research climate, supervision, and methodological patterns

This section addresses RQ2, focusing on how methodological designs and institutional factors such as research climate and supervision shape what becomes empirically visible in studies of research misconduct. This subsection synthesises how empirical studies conceptualise and investigate the conditions under which misconduct and questionable research practices emerge. Rather than focusing on specific behaviours, it examines the explanatory frameworks, measurement strategies, and methodological regularities that shape the empirical visibility of integrity problems.

Across the reviewed literature, research climate functions as a key integrative concept. It is typically operationalised through perceptions of fairness, openness, collegiality, supervision quality, and institutional support. Studies adopting this perspective tend to treat misconduct and QRPs not as isolated acts, but as outcomes of everyday working environments. Importantly, climate-based approaches often explain variation in self-reported misbehaviour more effectively than individual characteristics such as career stage or discipline (e.g., [13, 34, 38]). This reinforces the shift from attributing integrity failures to personal deficits towards understanding them as embedded in organisational contexts.

Supervision occupies a central position within this explanatory landscape. In the context of research integrity studies, supervision typically refers to mentoring relationships and organisational practices through which senior researchers structure guidance, oversight, and responsibility within research teams. Where measured explicitly, it is most often captured through indirect indicators, including clarity of expectations, availability of guidance, feedback practices, and oversight of data management. Weak or inconsistent supervision is repeatedly identified as a facilitating condition for questionable practices, particularly among early-career researchers. However, supervision is rarely examined from multiple perspectives simultaneously. Most studies rely on researchers’ accounts of being supervised, while comparatively few include supervisors themselves as respondents. This asymmetry limits empirical insight into how supervisory responsibilities are interpreted, enacted, or constrained within institutions.

Methodologically, the literature exhibits a strong alignment between respondent populations and data collection strategies. Survey-based designs dominate studies of researchers, faculty members, and doctoral candidates, enabling broad coverage but constraining the depth at which relational and contextual dynamics can be explored. By contrast, interview-based approaches are more commonly used when studying editors, research integrity officers, or ethics-related stakeholders. This pattern reflects practical considerations of access and sample size, but it also produces systematic differences in what kinds of knowledge are generated about different actors within the research system (e.g., [18, 37]). As a result, the voices of those subject to integrity pressures are captured primarily through standardised instruments, while those in governance roles are examined through more interpretive, qualitative lenses.

Several recurring methodological limitations follow from these patterns. The heavy reliance on self-reported data raises persistent concerns about social desirability, underreporting, and selective disclosure, particularly for severe forms of misconduct. Cross-sectional designs dominate, offering limited insight into how changes in policies, incentives, or organisational arrangements affect research practices over time. Disciplinary coverage is uneven, with a concentration in biomedical and social science contexts and less systematic attention to fields with different publication cultures or collaborative structures. Finally, many studies provide limited methodological detail in titles and abstracts, complicating secondary analyses and comparative syntheses.

Taken together, the empirical literature constructs an understanding of misconduct and QRPs that is shaped as much by methodological choices as by substantive concerns. Climate and supervision emerge as central explanatory themes, yet they are often measured indirectly and from a narrow set of perspectives. The alignment between methods and respondent status further structures what forms of harm, responsibility, and agency become visible. Recognising these patterns is essential for interpreting existing evidence and for identifying where empirical approaches may need to evolve in order to capture the systemic dimensions of research integrity more fully. These methodological patterns help explain why empirical studies tend to capture perceptions of misconduct more readily than the institutional conditions that enable or constrain such behaviour. This imbalance has important implications for how research integrity risks are interpreted, as empirical evidence becomes more detailed about perceptions of misconduct than about the systemic conditions under which such behaviour emerges.

## Discussion

### Institutional implications of empirical research on misconduct

The findings of this review can be interpreted in relation to the two research questions guiding the analysis. Taken together, the results show that both the operationalisation of harm and the empirical visibility of institutional conditions are strongly shaped by prevailing methodological approaches in the literature. This review shows that empirical research on scientific misconduct and questionable research practices has become more coherent in its thematic focus but remains uneven in its explanatory reach. Over the past decade, the field has converged around survey- and interview-based approaches that capture researchers’ perceptions, attitudes, and self-reported experiences. As a result, we now have a relatively detailed picture of how empirical studies operationalise harm associated with misconduct and questionable research practices, and how perceptions of research climate and supervision shape researchers’ accounts of integrity problems. This perception-oriented literature represents a genuine empirical achievement. It has moved the discussion beyond isolated scandals and moralised narratives and has demonstrated that integrity problems are closely tied to shared expectations, supervision practices, and organisational environments rather than to individual deviance alone [11, 19].

However, the very features that make this body of work coherent also delimit what it can explain. The heavy reliance on self-reported perceptions means that much of what we know concerns how misconduct is experienced and interpreted by researchers, not how risk is structurally produced and distributed across research systems. Survey instruments are well suited to capturing norms, attitudes, and perceived prevalence, but they are less effective at rendering visible cumulative, relational, and institutional dynamics. As a consequence, empirical knowledge is strongest where problems are easily verbalised and weakest where risks emerge indirectly, through the interaction of incentives, power asymmetries, and organisational constraints.

These limitations become particularly salient when considering contexts that are underrepresented or only implicitly addressed in the literature. A substantial share of studies does not specify a geographical or institutional setting, implicitly treating misconduct and QRPs as context-independent phenomena. Where contexts are named, research is concentrated in relatively stable and well-institutionalised systems, predominantly in Europe and North America. By contrast, transition systems and countries characterised by institutional instability, rapid reform, or weak enforcement capacities appear only sporadically. This absence is analytically consequential. In such environments, formal integrity policies may coexist with informal practices that govern access to resources, authorship, supervision, and protection from sanctions, shaping research behaviour in ways that remain largely invisible to standardised instruments [19]. Instruments developed in stable settings may therefore systematically underestimate risk or misclassify practices that are locally normalised but structurally problematic.

A related blind spot concerns the way risk is conceptualised. Although many studies explicitly reject an individualised framing of misconduct, empirical operationalisation often remains centred on individual behaviours and choices. Structural factors, such as evaluation regimes, funding competition, hierarchical dependency, and limited oversight, are typically measured indirectly, if at all. As a result, systemic risk tends to be inferred from individual responses rather than analysed as an emergent property of research systems [33]. This makes it difficult to distinguish between environments in which misconduct is sporadic and environments in which conditions consistently enable questionable practices, even in the absence of overt rule-breaking.

Methodological patterns reinforce this tendency. The alignment between respondent populations and data collection methods shapes what kinds of integrity problems become visible. Researchers and doctoral candidates are primarily studied through surveys, producing broad but shallow accounts of experience, while governance actors are examined through interviews that allow richer contextualisation but limited generalisation. Longitudinal designs remain rare, constraining insight into how changes in policy, incentives, or organisational structure affect behaviour over time, even though such dynamics are central to understanding research culture as a moving target [36]. Emerging and organised forms of misconduct, including coordinated authorship manipulation or paper mill activity, remain largely absent from standard instruments, despite their growing prominence in editorial practice and investigative reporting [15, 24]. Taken together, these patterns suggest that current empirical approaches are better at documenting perceptions of harm than at estimating the conditions under which harm is likely to arise and persist.

An additional blind spot concerns practices aimed at manipulating evaluative indicators rather than research outputs themselves. While ranking systems, citation metrics, and performance indicators are widely acknowledged as powerful drivers of research behaviour, empirical studies of misconduct and QRPs rarely treat indicator-oriented manipulation as an object of analysis in its own right. Practices such as strategic self-citation, coordinated citation boosting, or targeted authorship strategies are typically discussed in bibliometric or policy-oriented literatures, but remain largely absent from survey-based integrity research [2, 25, 26, 31]. As a result, a class of questionable practices that is structurally induced by evaluation regimes and closely tied to institutional competition remains weakly integrated into empirical models of research integrity, despite its potential to distort both knowledge production and organisational behaviour.

These observations point to a broader implication for the field. Descriptive mapping and perception-oriented studies have been essential in establishing misconduct and QRPs as systemic phenomena linked to research climate and supervision. However, further progress requires a shift from description to explanation. Rather than asking only how researchers perceive misconduct or how frequently specific practices are reported, future work needs to address how different configurations of institutional conditions generate varying levels of integrity risk. This entails moving towards models that integrate individual perceptions with organisational structures, incentive systems, and contextual instability.

Risk modelling offers one possible pathway for such integration. By conceptualising misconduct and QRPs as outcomes of interacting factors, rather than as isolated acts, it becomes possible to compare systems not only in terms of reported behaviour, but also in terms of their vulnerability to integrity failures. Such an approach does not replace survey-based evidence; instead, it builds on it by situating perceptions within a broader analytical framework. For research systems facing rapid transformation or persistent instability, this shift is particularly important. Without it, empirical research risks reproducing a partial and context-blind understanding of misconduct, one that captures how problems are experienced but not why they recur.

From this perspective, the present review highlights both the strengths and the limits of current empirical knowledge. We now understand much about how integrity problems are perceived and normalised within research communities. What remains underdeveloped is a systematic account of how institutional conditions shape the distribution and intensity of risk across different contexts. Addressing this gap is essential if empirical research on misconduct is to inform not only awareness and training, but also structural interventions and policy design.

This review is subject to several *limitations*. First, it focuses exclusively on empirical studies using survey-based, interview-based, or mixed-methods designs. While appropriate for examining perception- and climate-oriented approaches, this scope excludes other empirical strands, such as bibliometric, retraction-based, experimental, or forensic analyses of misconduct and QRPs.

Second, the review is limited to English-language journal articles indexed in Scopus and the Web of Science Core Collection. This restriction enhances comparability but likely underrepresents national studies, policy reports, and grey literature, particularly from research systems that are less internationally visible.

Third, the review reflects the limitations of the underlying literature, which relies predominantly on self-reported data. Such data are susceptible to social desirability and underreporting, especially for sensitive or structurally embedded practices that are difficult to articulate in standardised instruments.

Finally, substantial heterogeneity in study designs and operationalisations constrains comparability and precludes quantitative synthesis. Accordingly, the findings should be interpreted as a structured mapping of empirical approaches and recurring blind spots, rather than as definitive estimates of prevalence or causality.

### Answering the research questions

- *RQ1: Operationalisation of harm.* The review shows that empirical studies typically operationalise harm through perception-based survey instruments that ask researchers to evaluate the severity of different forms of misconduct and questionable practices. While this approach allows comparisons across studies, it also introduces blind spots, particularly regarding practices that are embedded in everyday research routines and therefore remain difficult to measure directly.
- *RQ2: Institutional visibility and methodological design.* The analysis further demonstrates that methodological choices strongly shape what becomes visible in empirical studies of research misconduct. Survey-based designs tend to capture perceptions of behaviour and attitudes, whereas institutional conditions such as supervision structures, organisational incentives, and research climate are more difficult to operationalise systematically.

## Conclusion

This review set out to examine how scientific misconduct and questionable research practices are empirically studied within survey- and interview-based research published over the past decade. By synthesising 229 empirical studies, the review addressed two central questions: how harm is operationalised in research on misconduct and QRPs, and how methodological designs shape the visibility of institutional factors such as research climate and supervision. Rather than estimating prevalence or ranking the severity of individual behaviours, the analysis focused on how empirical studies operationalise harm and how methodological designs shape the visibility of institutional conditions such as research climate and supervision. The findings show that this body of literature has matured in important ways. Empirical research has largely moved beyond individualised and moralised accounts of misconduct, offering instead a nuanced picture of how perceptions, norms, supervision practices, and research climate shape everyday research behaviour.

Nevertheless, the review reveals clear structural limitations in how misconduct and QRPs are currently investigated. Empirical attention is concentrated on self-reported perceptions and attitudes, while the institutional conditions that generate and sustain integrity risks remain weakly operationalised. Contextual variation is unevenly addressed, with research predominantly focused on stable and well-institutionalised systems and limited engagement with transition contexts or environments characterised by institutional instability. As a result, much of what is empirically documented concerns how integrity problems are experienced, rather than how risk is produced, distributed, and reproduced across research systems.

These patterns have important implications for both research and policy. Perception-oriented studies provide valuable insight into local norms and climates, but they offer limited guidance for identifying environments in which questionable practices are likely to emerge or persist. Without analytical frameworks that explicitly integrate organisational structures, incentive regimes, and governance capacities, empirical evidence risks remaining descriptive, even when it adopts a systemic vocabulary.

Future research would therefore benefit from complementing perception-based approaches with models that treat misconduct and QRPs as outcomes of interacting institutional factors rather than isolated behaviours. Risk-oriented analytical frameworks offer a promising direction in this respect, particularly for comparing research systems that differ in stability, oversight, and evaluative pressure. Such approaches can build on existing empirical knowledge while addressing its current blind spots.

In this sense, the present review contributes not only a structured mapping of empirical approaches to scientific misconduct and questionable research practices, but also a clearer understanding of their limits. Recognising what current evidence can and cannot explain is a necessary step towards developing more robust analytical tools capable of informing institutional reform and evidence-based research integrity policy.

## Data Availability

The dataset underlying this study, containing metadata for the 229 empirical studies included in the review, is openly available on Zenodo at 10.5281/zenodo.18096483.
